# Geographical variations and determinants of iron and folic acid supplementation during pregnancy in Ethiopia: analysis of 2019 mini demographic and health survey

**DOI:** 10.1186/s12884-022-04461-0

**Published:** 2022-02-16

**Authors:** Koku Sisay Tamirat, Fentahun Bikale Kebede, Tajebew Zayede Gonete, Getayneh Antehunegn Tessema, Zemenu Tadesse Tessema

**Affiliations:** 1 Department of Epidemiology and Biostatistics, Institute of Public Health, College of Medicine and Health Sciences, University of Gondar, Gondar, Ethiopia; 2 Ethiopia Public Health Institute, Addis Ababa, Ethiopia; 3 Department of Health System and Policy, Institute of Public Health, College of Medicine and Health Sciences, University of Gondar, Gondar, Ethiopia

**Keywords:** Iron and folic acid, Geographical variations, Pregnant women, Ethiopia

## Abstract

**Background:**

One of the packages of critical antenatal care treatments for pregnant women includes iron and folic acid (Fe/FA) supplementation. Using recently available and nationwide representative survey data, this study aimed to determine the spatial patterns and drivers of Fe/FA supplementation during pregnancy.

**Method:**

The data for this study was obtained from Ethiopia’s 2019 Mini Demographic and Health Survey (EMDHS). We used the Kid’s Record (KR) dataset, and a total weighted sample of 3926 reproductive-age women who gave birth within the previous 5 years was used as the study’s final sample size. To analyze the spatial distributions (geographic variation of Fe/FA supplementation) different statistical software like Excel, ArcGIS, and Stata 14 were used. A two-level multilevel binary logistic regression model was fitted to identify both individual and community-level factors associated with Fe/FA supplementation during pregnancy.

**Result:**

This study found that there were significant geographical variations of iron and folic acid supplementation across Ethiopia, eastern and southern parts of the country were predicted to have low Fe/FA supplementation coverage. Advanced maternal age (AOR = 0.75: 95%CI: 0.59 0.96), resides in developing region (AOR = 0.57, 95%CI: 0.43 0.74), not attended formal education (AOR = 0.60, 95%CI: 0.39 0.92), middle (AOR = 1.51, 95%CI: 18 1.93) and rich wealth status (AOR = 1.48, 95%CI: 1.15 1.91), and four and above ANC visits (AOR = 4.35 95%CI: 3.64 5.21) were determinants of iron and folic acid supplementation among pregnant women.

**Conclusion:**

Our research found that there were geographical variations across the country, with low coverage seen in Ethiopia’s eastern and southern regions. Iron and folic acid supplementation coverage were inadequate among pregnant women with low education, advanced maternal age, and those from underdeveloped countries. Conversely, increasing iron and folic acid uptake was associated with higher socioeconomic class and four or more ANC visits. The findings of this study highlight the importance of increasing maternal health care, such as iron and folic acid supplements, for underserved populations.

## Background

Because of their altered physiological condition, increased blood losses, higher vitamin requirements, and poor feeding habits or nutritional status, pregnant women are more vulnerable to anemia than other groups. Anemia affects a quarter of the population and 41.8% of pregnant mothers worldwide and accounts for one-fifth of all maternal deaths [1]. The burden is carried mostly by third-world countries, particularly Africa, which has a maternal prevalence of 55.8% and is more susceptible to helminthic infestation and malaria infection [1, 2].

Iron deficiency is the leading cause of anemia in pregnant women, according to the 2016 Ethiopia Demography and Health Survey (EDHS) [3]. Cross-sectional research in Ethiopia’s Oromia region found that 19.3% of pregnant women had iron deficiency anemia (IDA) [4]. Furthermore, anemia in pregnant women contributes to maternal mortality; a study by Young et al. found a direct link between anemia and maternal deaths, with each 10 g/L rise in hemoglobin associated with a 29% reduction in maternal mortality [5, 6]. Anemia during pregnancy causes maternal mortality and morbidity, as well as child morbidity and mortality, as well as a reduction in women’s quality of life, job capability, and physical performance [7, 8]. As a result, the World Health Assembly set a goal of halving the prevalence of anemia in women of reproductive age by the end of 2025 compared to 2010 [1]. To prevent maternal anemia, puerperal sepsis, low birth weight, and preterm birth, the World Health Organization (WHO) recommends daily oral Fe/FA supplementation for pregnant women [9].

Some of the primary interventions used to lower the incidence of anemia during pregnancy include counseling on healthy eating and nutritional diversity, promotion of insecticide-treated bed nets (ITN) for malaria management, and deworming during pregnancy [10]. According to the Ethiopia Demographic and Health Survey report, despite all recommended measures, the prevalence of anemia during pregnancy increased from 22 to 29.1% between 2011 and 2016 [3, 11]. Another study in the same setting found that only 60% of pregnant women received Fe/FA supplementation, with a high rate of non-adherence [6, 12, 13]. The World Health Organization (WHO) recommends Fe/FA supplementation for women who are pregnant for 90 days or more [10]. Meanwhile, iron and folic acid supplementation reduced maternal anemia, newborn mortality, and low birth weight significantly [5, 8]. Furthermore, Fe/FA supplementation is a predictor of maternal health care quality, medicine availability, and the general soundness of the country’s health care system [14].

Many studies have been undertaken among pregnant women to discover the main factors linked to poor iron and folic acid adherence during pregnancy [12, 13, 15, 16]. Furthermore, the investigations were conducted in a single institution or a small geographic area, making them unrepresentative. Unfortunately, there is insufficient evidence about geographic distributions, and hotspot areas of high and low Fe/FA supplementation coverage in the research context have yet to be found. While, geographical analysis studies would provide information regarding hotspot areas for prioritizing, resource allocation, and prediction of healthcare utilization trends.

Therefore, this study aimed to assess the spatial distributions and determinants of iron/folic acids supplementation during pregnancy using the recently available and representative data from the national census. Finding from this study could help to design appropriate interventions and targeted allocation of resources [17]. Moreover, the finding of this study could give an insight into the quality of maternal health services. Iron supplementation is one of the antenatal care services offered to pregnant women, therefore non-compliance with WHO recommendations could undermine the quality of care.

## Methods

### Data source

The 2019 Ethiopia Mini Demographic and Health Survey (EMDHS) provided the data for this study. In the years 2000, 2005, 2011, and 2016, four full-scale DHS surveys were conducted. The first EMDHS took place in 2014, and the second took place in 2019. The data was collected between March 21 and June 28, 2019. The EMDHS is a national survey that gathers information about men, women, and children. Of which, KR datasets were used in this investigation. The mini EDHS utilizes a two-staged sampling procedure to gather data from 9 Regional States and two City Admirations. In the first stage, a total of 305 EAs (93 in urban areas and 212 in rural areas) were selected with probability proportional to EA size (based on the 2019 PHC frame) and with independent selection in each sampling stratum. In the second stage, lists of households served as a sampling frame for the selection of households for detail you can find elsewhere [18]. Individual sample weight (v005/1,000,000) was used in all analyses to account for over-and under-sampling. Using data from the KR dataset a total weighted sample of 3926 women of the reproductive age group was considered as the sample size for this study. For this study, 8885 women were interviewed throughout the survey, of whom 5846 women ever gave birth and 3979 women who gave birth in the preceding 5 years before the survey. The dataset is available in the public domain and can be downloaded from https://dhsprogram.com/data/available-datasets.

### Outcome variable

Iron folic acid supplementation was used as the study’s outcome variable. This was determined by asking respondents if they had taken iron and folic acid supplements during their pregnancy in the 5 years before the study. For this research, we used the Kids Record (KR) file, which contains information about recent maternal and pregnancy-related behaviors and features. The response categories for the variable were “Yes” and “No,” which were coded as “1” and “0,” respectively.

### Independent variables

Individual and community-level characteristics were examined in our study based on theoretical and practical significance as well as the availability of variables in the dataset. The factors were chosen based on their associations with iron and folic acid supplementation coverage in previous Ethiopian investigations [2, 16, 19–22].

### Individual-level factors

The individual-level variable included in this study was the age of women, level of education, wealth status, marital status, sex of household head, mode of delivery, and parity [2, 16, 19–22].

### Community-level factors

The Community level variables included in this study were the place of residence and the region from which the samples drown.

### Statistical analyses

We employed both spatial and multilevel analyses in analyzing the data, the result presented in the form of tables, texts, graphs, and maps.

### Spatial analysis

To analyze the spatial distribution (geographic variation of folic acid supplementation) different statistical software like Excel, ArcGIS, and Stata 14 were used. The weighted frequency of outcome variable (coverage of Iron folic acid supplementation with the response (yes/no) with cluster number and geographic coordinate data was merged in Stata 14. Among 305 clusters 93 were in urban areas and 212 in rural areas. The data was exported to CSV delimited format to make ready data for ArcGIS 10.7 for spatial analysis.

### Spatial autocorrelation analysis

To check whether there is a clustering effect in coverage of iron and folic acid supplementation in Ethiopia, a spatial autocorrelation analysis was done. This analysis result gives Global Moran’s I value, Z-score, and *P*-value for deciding whether the data is dispersed or random, or clustered. Moran’s I value close to positive 1 indicates there is a clustering effect, close to negative one indicates dispersed and close to zero random. If *P*-value is significant and Moran I value is close to mean that coverage of iron and folic acid supplementation had a clustering effect.

### Hot spot analysis (Getis-OrdGi* statistic)

The hot spot analysis tool gives a Getis_Ord or Gi* statistics for a cluster in the dataset. Statistical values like Z-score and *p*-value is computed to determine the statistical significance of clusters. Results of the analysis with high GI* value means hot spot areas (high coverage of Iron and folic acid supplementation) and low GI* value means cold spot areas (low coverage of Iron and folic acid supplementation).

### Spatial interpolation or prediction

Spatial prediction is one of the techniques of furcating unsampled areas based on sampled areas. In Ethiopia for this EMDHS, a total of 305 clusters were selected to take a sample for this area that is believed to be representative of the country. Based on 305 sample areas it is possible to predict the remaining parts of Ethiopia. Thus, Ordinary Kriging prediction methods were used for this study to predict coverage of Iron folic acid supplementation in unobserved areas of Ethiopia.

### Multilevel analysis

A two-level multilevel binary logistic regression model was fitted to evaluate the individual and community level factors associated with iron and folic acid supplementation in Ethiopia. In the modeling, women were nested within the community, then community was nested within clusters. To account for the unexplained variability at the community level, clusters were proposed as random effects. A total of four models were fitted. Firstly, we fitted an empty model, model I, which contained no predictors (random intercept). Following that, model II only included individual-level variables, model III only included community-level variables, and model IV included both individual-level and community-level variables. The odds ratio and related 95% confidence intervals were provided for all models. These models were fitted by a Stata command “melogit” for the identification factors associated with coverage of Iron folic acid supplementation in Ethiopia. The Intra-class Correlation Coefficient (ICC), the Median Odds Ratio (MOR), and the Proportional Change in Variance (PCV) were computed to assess the clustering effect/variability. The log-likelihood ratio (LLR), Akaike Information Criteria (AIC) measure, and Schwarz’s Bayesian Information Criteria (BIC) were used to compare models. The best fit model has the highest log-likelihood and the lowest AIC.

## Result

### Socio-demographic characteristics

The median age of women was 28 years with IQR of 24 to 33 years, nearly one-third (30.8%) of women aged between 25 and 29 years. About 74.8, 51.9, 47.1% were rural dwellers, had no formal education, and had poor socio-economic conditions, respectively. Meanwhile, about 31.8and 46.8% were orthodox and Muslim religion followers. About 20% of households were headed by females (Table 1).

**Table 1:** Socio-demographic characteristics of women in Ethiopia

Characteristics	Weighted frequency (N)	Weighted percentage (%)
**Age of women**		
Less than 24 years	996	25.4
25–34 years	1991	50.7
Above 35 years	938	23.9
**Residence**		
Urban	1026	26.1
Rural	2900	73.9
**Marital status**		
Married	3684	93.8
Divorced /widowed/ separated	241	6.2
**Level of education**		
No formal education	2014	51.3
Primary	1414	36
Secondary	344	8.8
Diploma and above	152	3.9
**Region**		
Tigray	286	7.3
Afar	51	1.3
Amhara	839	21.4
Oromia	1519	38.7
Somalia	218	5.5
Benishangual Gumuz	47	1.2
SNNPR	787	20
Gambella	19	0.5
Harari	11	0.3
Addis Ababa	126	3.2
Dire dawa	21	0.5
**Wealth status**		
Poor	1647	41.9
Middle	761	19.4
Rich	1518	38.7
**Sex of household head**		
Male	3401	86.6
Female	525	13.4
**Religion**		
Orthodox Christian	1440	36.7
Protestant	1082	27.6
Muslim	1339	34.1
Other	63	1.6

### Maternal health and reproductive characteristics

About 61.5% of women were primiparous and the remaining were multiparous, the median age of women at firth birth was 18 years with IQR of 16 to 24 years. About 54.8% of women gave birth in the health facilities, about 73.8% of women had antenatal care follow up to the recent pregnancies, and nearly half (49.7%) women booked first antenatal check during second trimesters of pregnancies. Nurses, doctors, health extension workers, and public health officers were the commonest service providers of antenatal care visits (Table 2).

**Table 2:** Reproductive and maternal health services utilization of women in Ethiopia

Characteristics	Weighted frequency (N)	Weighted percentage (%)
**ANC follow up**		
Yes	2923	74.4
No	1003	25.6
**First ANC booking**		
First trimester	1092	37.4
Second trimester	1563	53.5
Third trimester	267	9.1
**Number of ANC follow-up**		
Less than 4	2228	56.7
Above 4	1698	43.3
**ANC service provider**		
Nurses	1073	27.3
Midwives	1005	25.6
Health extension workers	646	16.5
Doctors	310	7.9
Health officers	277	7
**Place of delivery**		
Home	1864	47.5
Health facility	2062	52.5
**Parity**		
Primiparous	2505	63.8
Multiparous	1420	36.2
**Birth order of the recent birth**		
1 to 2	1562	39.8
3 to 4	999	25.5
Above 4	1363	34.7
**Under-five child mortality**		
Yes	156	4
No	3770	96

**Fig. 1:**
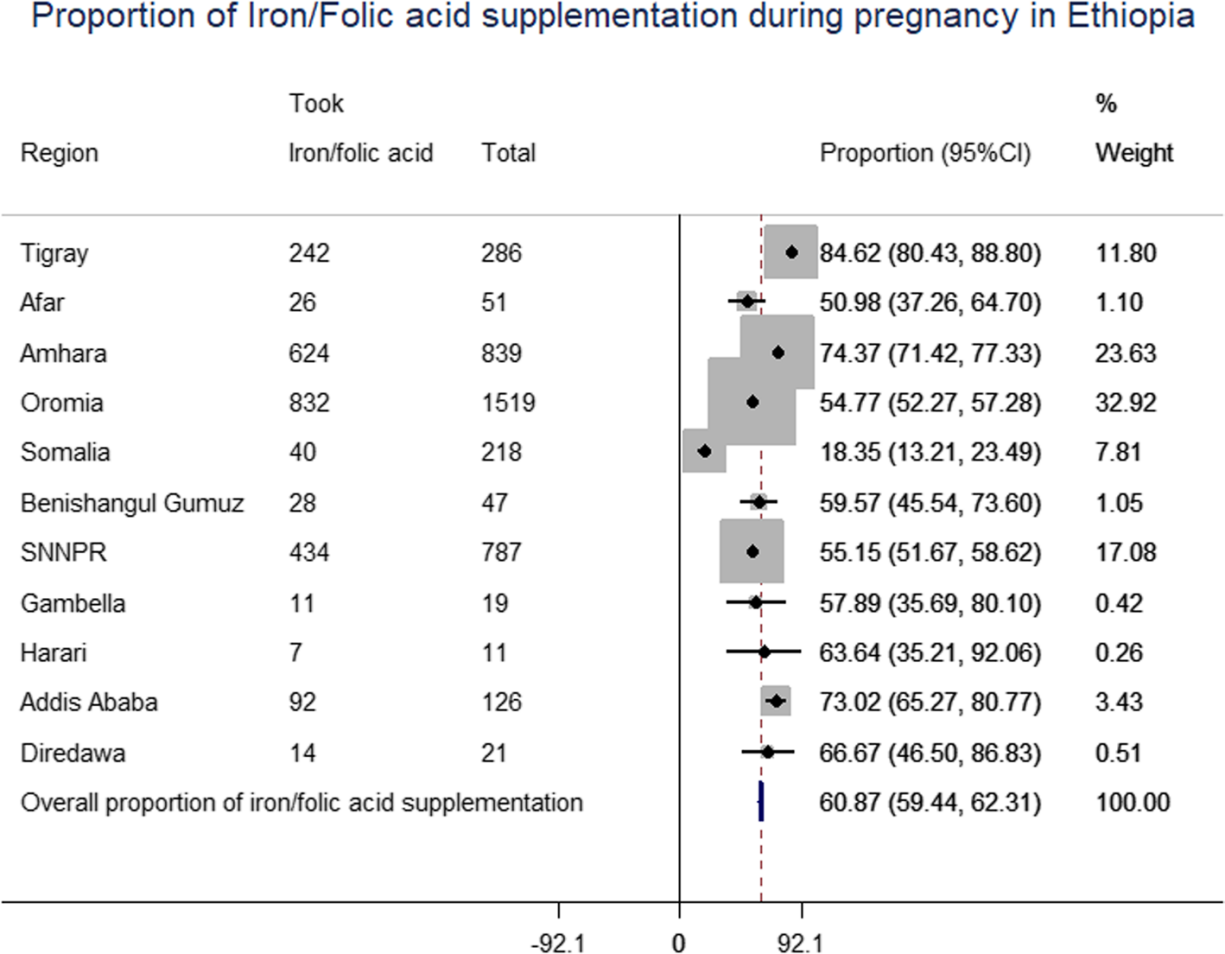
Forest plot of proportion of iron and folic acid supplementation

**Fig. 2:**
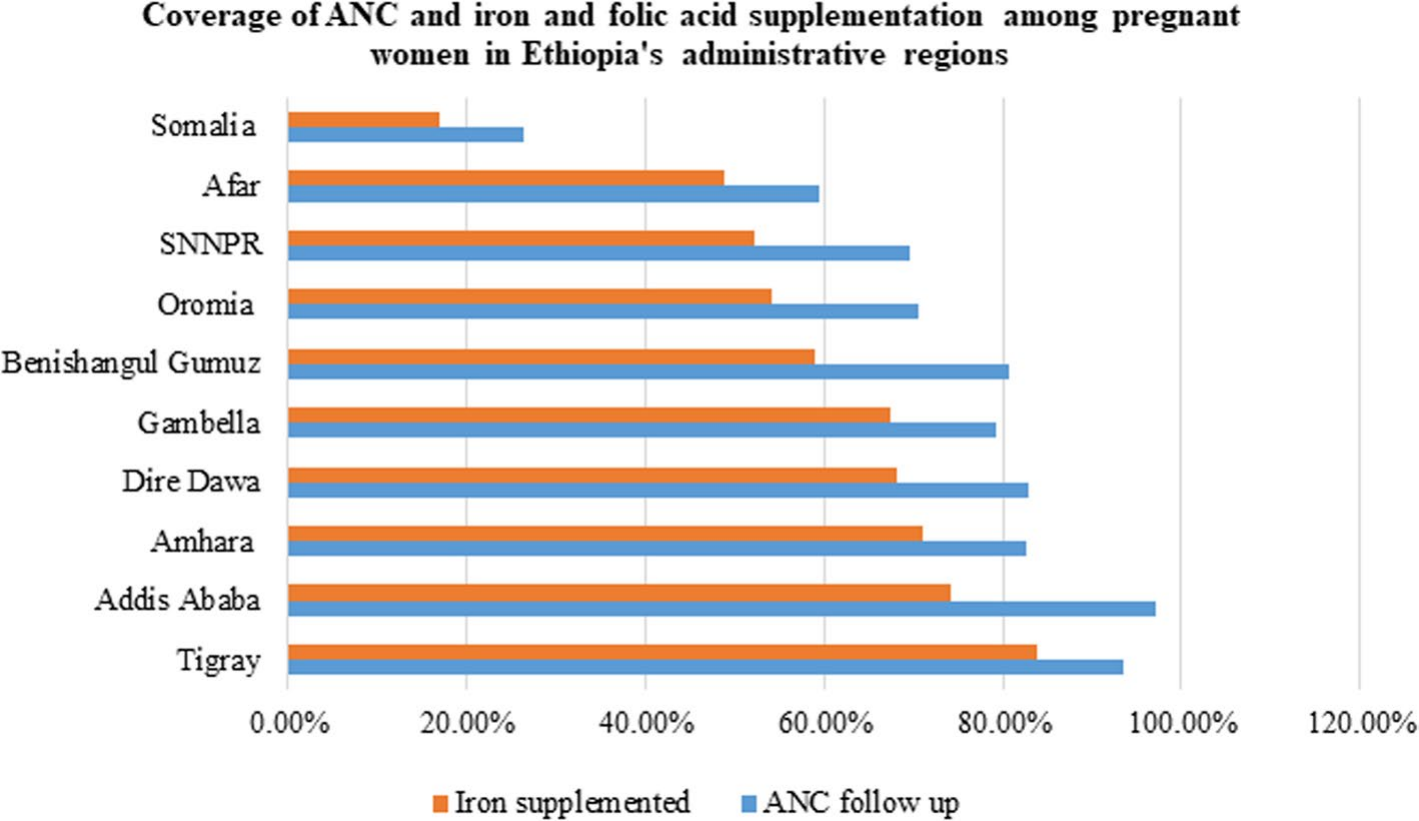
Coverage of ANC and Fe/FA among study participants in Administrative region of Ethiopia

About 60% of pregnant women during the 5 years preceding the survey took Fe/FA supplementation, of whom only 20% of them took for 90 days and above (Fig. 1). More specifically, the coverage of iron and folic acid supplementation ranged from 83.8% in the Tigray region to 18% in Ethiopia Somalia region. About 58.8% of pregnant women from poor households had ANC follow up of which 44.5% of them obtained iron and folic acid supplementation which was quite lower than women from rich households (Fig. 2).

### Spatial distributions of iron and folic acid supplementation during pregnancy in Ethiopia

**Fig. 3:**
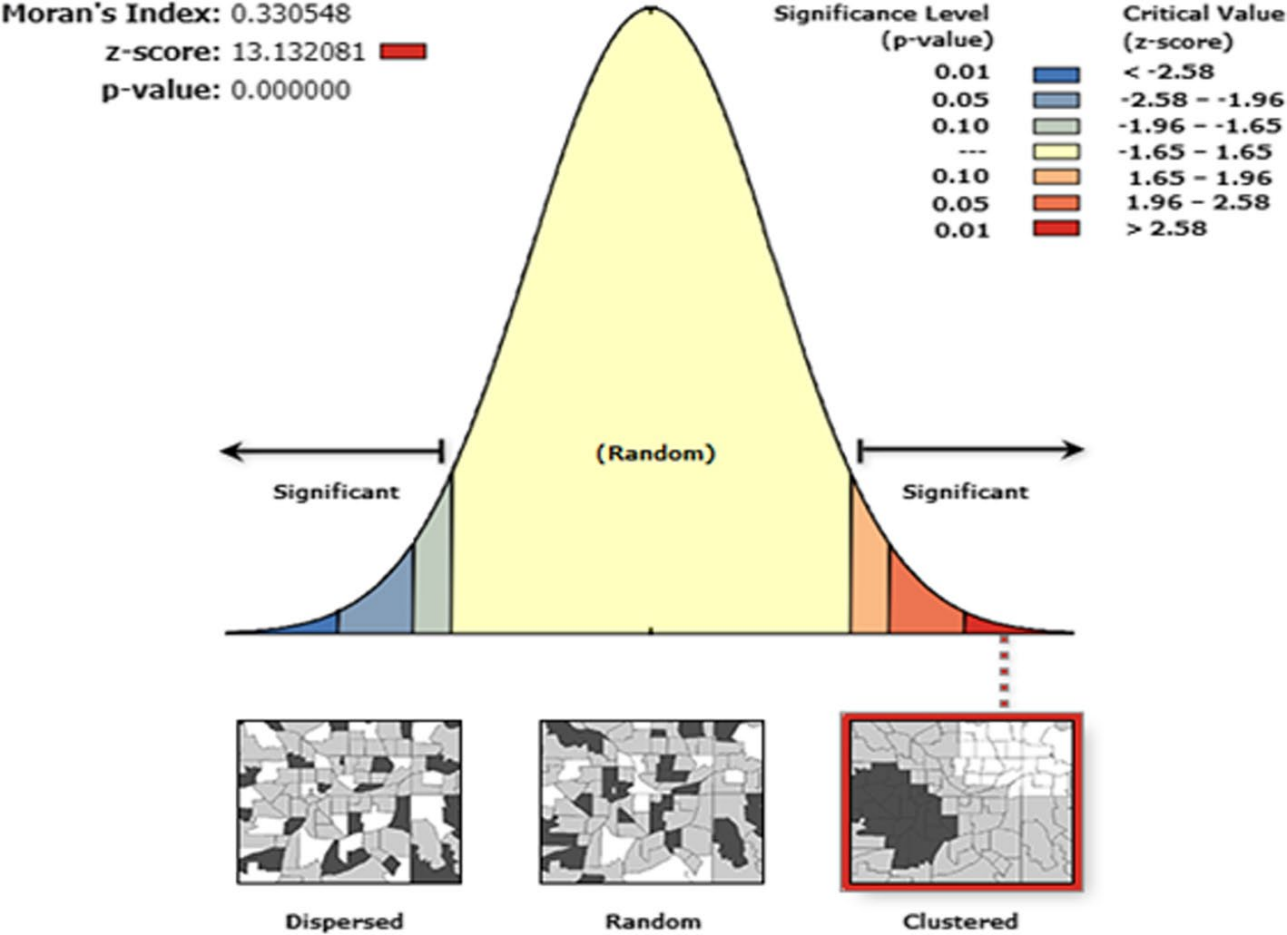
Spatial autocorrelation

**Fig. 4:**
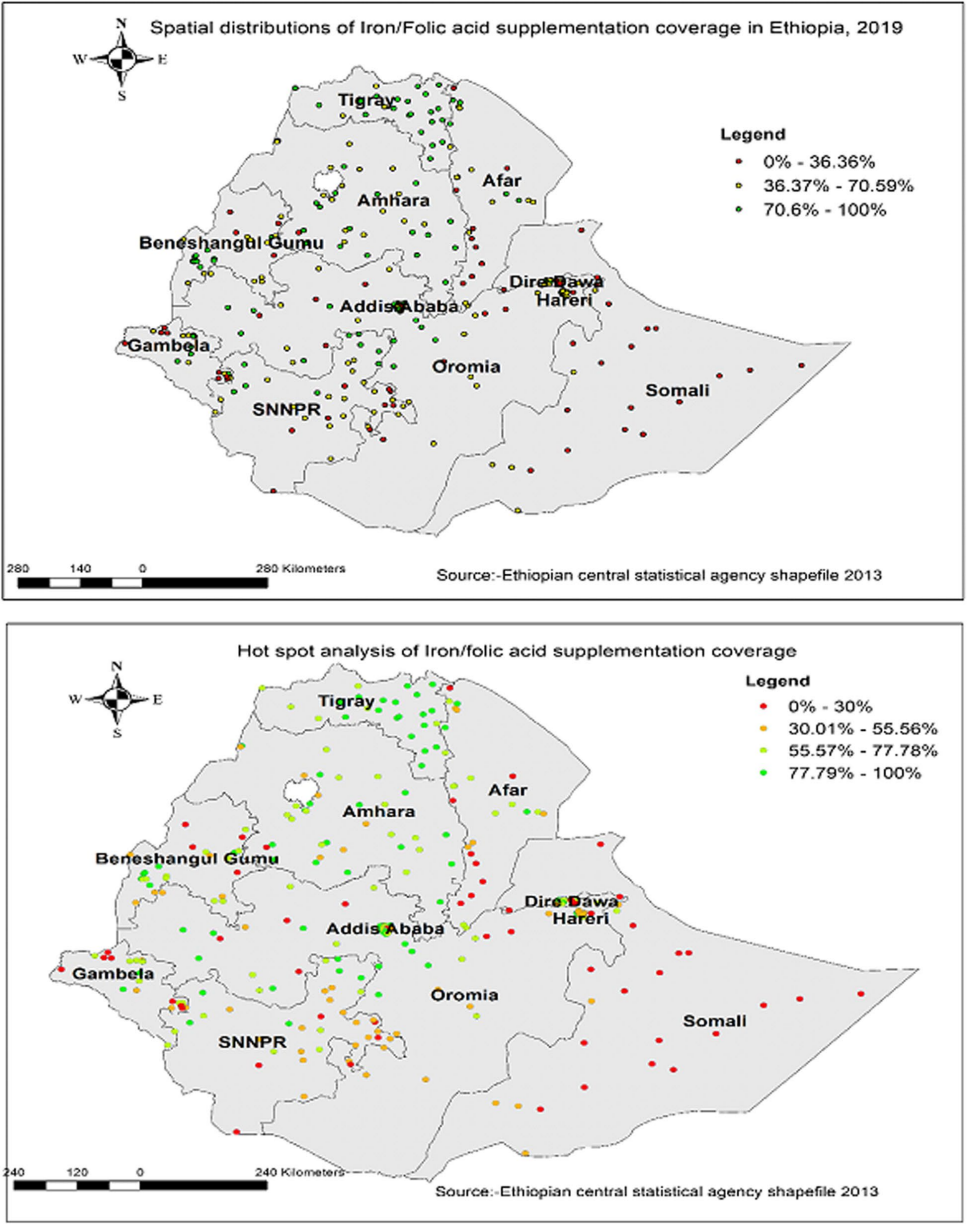
Spatial distribution and hot spot analysis of iron and folic acid supplementation in Ethiopia

**Fig. 5:**
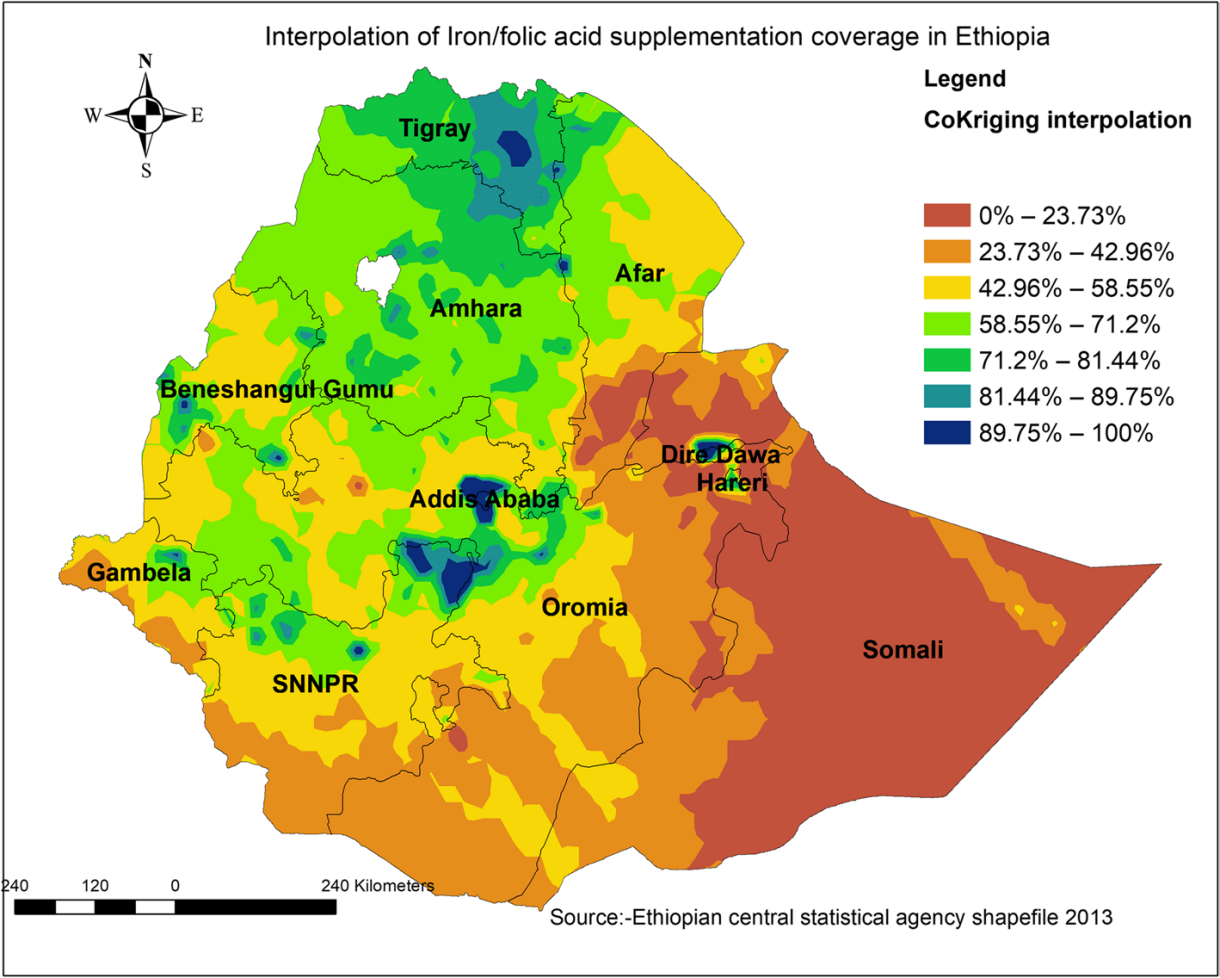
Spatial interpolation of iron folic acid supplementation in Ethiopia

Spatial analysis showed that the global Moran’S index value of 0.33 with a *p*-value of less than 0.05 reflects non-random distribution of Fe/FA supplementation coverage, given that the central and northern part of Ethiopia had better coverage of Fe/FA supplementation (Figs. 3 and 4). Whereas, Easter part of Ethiopia Afar, Somalia, and some southern Ethiopia had lower coverage of Fe/FA supplementation. On the other hand, hot spot analysis showed that the majority of Somalia, Afar, and some part southern parts of the country were identified hotspot areas of low coverage (Fig. 4). Likewise, the eastern and southern part of the country has been predicted to have low coverage of Fe/FA supplementation based on Kriging interpolation findings. On the other hand, the Northern and central parts of Ethiopia would have high coverage of supplementation (Fig. 5).

### Determinants of iron and folic acid supplementation during pregnancy

Pregnant women aged above 35 years had 25% lower odds of iron and folic acid supplementation compared to those younger than 24 years (AOR = 0.75, 95%CI: 0.59 0.96). Similarly, for pregnant women who didn’t attend formal education, the odds of iron and folic acid supplementation decreased by 40% than those who attended diploma and higher education (AOR = 0.60, 95%C: 0.39 0.92). Likewise, those pregnant women living in developing regions the odds iron and folic acid supplementation decreased by 43% than those living in developed regions (AOR = 0.57, 95%CI: 0.43 0.74). On other hand, pregnant women from the middle had 1.51 (AOR = 1.51, 95%CI: 1.18 1.93) and rich economic status had 1.48 (AOR = 1.48, 1.15 1.91) times higher odds of iron and folic acid supplementation than poorer pregnant women. Meanwhile, for Pregnant women who had attended four and above antenatal care visits, the odds of iron and folic acid supplementation was 4.35 times higher than women with few visits (AOR = 4.35, 95%CI: 3.64 5.21) (Table 3).

**Table 3:** Multilevel logistic regression analysis of Iron/folic acid supplementation during pregnancy in Ethiopia

Characteristics	Model (I) AOR 95%CI (without covariate)	Model (II) AOR 9%CI (community-level factors)	Model (III) (individual- level factors)	Model (IV) AOR with 95%CI (individual and community level factors)
**Region**				
Developed region		Ref		Ref
Developing region		0.40(0.30 0.54)		0.57(0.43 0.74)*
**Residence**				
Rural		0.40(0.29 0.56)		0.90(0.64 1.27)
Urban		Ref		Ref
**Age of women**				
Less than 24			Ref	Ref
25–34 years			1.09(0.89 1.32)	1.08(0.89 1.32)
Above 35 years			0.76(0.60 0.97)	0.75(0.59 0.96)*
**Marital status**				
Married			1.16(0.85 1.60)	1.20(0.87 1.64)
Divorced/ separated/ widowed			Ref	Ref
**Parity**				
Primiparous			Ref	Ref
Multiparous			0.87(0.74 1.03)	0.92(0.77 1.08)
**Level of education**				
No formal education			0.58(0.38 0.88)	0.60(0.39 0.92)*
Primary education			0.89(0.59 1.34)	0.92(0.61 1.39)
Secondary education			1.28(0.80 2.03)	1.30(0.82 2.07)
Diploma and above			Ref	Ref
**Wealth status**				
Poor			Ref	Ref
Middle			1.56(1.23 1.99)	1.51(1.18 1.93)*
Rich			1.61(1.27 2.03)	1.48(1.15 1.91)*
**Sex of house hold head**				
Male			Ref	Ref
Female			1.02(0.82 1.28)	1.06(0.84 1.33)
**Number of ANC visits**				
Less than 4 visits			Ref	Ref
4 and above visits			4.57(3.82 5.46)	4.35(3.64 5.21)*
**Random variation (effect variation), i.e measure of variation for iron/folic acid supplementation**				
Community level variance (SE)				
*P*-value	< 0.0001	< 0.0001	< 0.0001	< 0.0001
Deviance	4835.542	4765.26	4373.049	4354.999
ICC%	32.2	25.7	20.1	18.9
PCV%	Reference	36.8%	46.9%	50.8%
MOR (95%CI)	3.27(2.86 3.81)	2.75(2.43 3.16)	2.35(2.03 2.75)	2.26(1.95 2.62)

*CI* Confidence Interval, *ICC* Intra-Class Correlation, *MOR* Median Odds Ratio, *PCV* Proportion of Change in Variance, *Ref* References

* Shows statistical significance at 0.05 *P*-value

## Discussion

According to the findings, over 60% of reproductive-age women took Fe/FA supplements during their most recent pregnancy, but only 20% of pregnant women took iron for 90 pills or more. The spatial distribution of Fe/FA supplementation was significantly different across regions, with hotspot areas of high Fe/FA supplementation coverage identified in the central and northern parts of the country, and cold spot areas (low iron and folic acid supplementation coverage) identified in the eastern and southern parts. This finding was consistent with a previous study in Ethiopia [23, 24]. Fe/FA supplementation in Ethiopia’s eastern and southern regions can be related to the country’s underdeveloped and fragile health system, which offers little maternal health services such as prenatal care. Furthermore, pastoralists in the eastern and southern parts of Ethiopia are frequently without a fixed domicile, making it difficult to provide integrated maternal healthcare services, such as iron and folic acid supplements during pregnancy [25]. As a result, further interventions are needed to increase iron/folic acid coverage through the availability of medications and the use of technology in hard-to-reach places. Establishing mobile and satellite clinics to provide crucial health services to vulnerable groups such as pregnant women and children in pastoralist communities that are difficult to reach [26].

Furthermore, Fe/FA supplementation was found to be influenced by the area, household wealth level, women’s age, level of education, and the frequency of ANC visits. As a result, pregnant women living in developing regions had reduced coverage of Fe/FA supplementation throughout pregnancy. This result was consistent with the results of the spatial analysis, as shown in Fig. 3. Meanwhile, those emerging regions have a poor health system and insufficient coverage of maternal health services such as ANC. Furthermore, as seen in Fig. 1, those developing regions had a low proportion of women attending ANC visits, which is in line with previous research [27].

Women who didn’t attend formal education had lower odds of iron and folic acid supplementation than women who had a diploma and above levels of education. This finding was consistent with the results of previous studies [6]. This could be due to the fact increased maternal level of education results in the corresponding improvement in health-seeking behavior during pregnancy including iron and folic acid supplementation.

On the other hand, women who were in the middle and rich socioeconomic status were associated with increased odds of iron/folic acid supplementation coverage. This could be because women having better economic status have improved access to health services and can afford medication costs [6, 12, 13]. Thus, alternative strategies such as improving health insurance coverage to avoid catastrophic expenses and making basic maternal health services free of charge may improve uptake of maternal health services [28]. Advanced maternal age (above 35 years) during pregnancy is associated with lower odds of Fe/FA supplementation than younger pregnant women. This could be because women in the older age group had lower uptake of maternal health services like ANC. This finding was supported by previous studies by *Tamirat* et al. in Ethiopia which showed women aged 35–49 years faced higher challenges of health care accesses [29].

Pregnant women who had four and above antenatal care visits were associated with higher odds of iron and folic acid supplementation. This finding could be attributed to the increased uptake of antenatal care gives the opportunity for Fe/FA supplementation [13, 15]. Moreover, ANC visit is the entry point for the utilization of maternal health care services and it is the key opportunity for nutritional counseling services, and nutritional supplementation.

This research has implications for pregnant women, health care personnel, and health care planners in terms of improving iron and folic acid supplements. Furthermore, the findings of this study may help improve pregnancy and birth outcomes caused by anemia issues. Furthermore, hotspot locations discovered may provide insight into targeted Fe/FA supplementation among expectant women. Despite the advantages of nationally representative data and geographic regions, this study has some weaknesses. For starters, secondary data sources lack maternal clinical and health facility features. Second, features of newborns such as birth weight and hemoglobin level were not captured and presented in the dataset. Furthermore, for the sake of privacy, the geographic locations (GPs) of enumeration areas were displaced up to 2 km in urban areas, 5 km for most enumeration areas in rural areas, and 10 km for 1% of clusters in rural areas, which may have an impact on the estimated cluster effects in the spatial regression.

## Conclusion

This research revealed unequal geographic distributions of iron and folic acid supplementation across Ethiopia, with low coverage in the east and south. Iron and folic acid supplementation coverage were low among pregnant women with low education, advanced maternal age, and those from underdeveloped countries. Conversely, increasing iron and folic acid usage was associated with higher socioeconomic status and four or more ANC visits. The findings of this study highlight the importance of increasing maternal health care, such as iron and folic acid supplements, for underserved populations.

## Data Availability

The datasets used during the current study are available from the corresponding author.

## Abbreviations

ABO: Adverse Birth Outcomes
ANC: Antenatal Care
AOR: Adjusted Odds Ratio
CI: Confidence Interval
DHS: Demographic and Health Survey
EA: Enumeration Area
Fe/FA: Iron and Folic acid
LBW: Low Birth Weight
LLR: Likelihood Ratio
SD: Standard Deviation
SGD: Sustainable Development Goal
SSA: Sub-Saharan Africa
